# P-tau217 as a Reliable Blood-Based Marker of Alzheimer’s Disease

**DOI:** 10.3390/biomedicines12081836

**Published:** 2024-08-13

**Authors:** Roy Lai, Brenden Li, Ram Bishnoi

**Affiliations:** 1 Morsani College of Medicine, University of South Florida, Tampa, FL 33612, USAbli20@usf.edu (B.L.); 2 Department of Psychiatry and Behavioral Neurosciences, University of South Florida, Tampa, FL 33613, USA; 3 USF Health Byrd Alzheimer’s Center and Research Institute, Tampa, FL 33613, USA; 4 USF Memory Disorder Clinic, Tampa, FL 33613, USA

**Keywords:** Alzheimer’s disease, biomarkers, tau, p-tau217, clinical utility

## Abstract

Amyloid plaques and tau tangles are the hallmark pathologic features of Alzheimer’s disease (AD). Traditionally, these changes are identified in vivo via cerebrospinal fluid (CSF) analysis or positron emission tomography (PET) scans. However, these methods are invasive, expensive, and resource-intensive. To address these limitations, there has been ongoing research over the past decade to identify blood-based markers for AD. Despite the challenges posed by their extremely low concentrations, recent advances in mass spectrometry and immunoassay techniques have made it feasible to detect these blood markers of amyloid and tau deposition. Phosphorylated tau (p-tau) has shown greater promise in reflecting amyloid pathology as evidenced by CSF and PET positivity. Various isoforms of p-tau, distinguished by their differential phosphorylation sites, have been recognized for their ability to identify amyloid-positive individuals. Notable examples include p-tau181, p-tau217, and p-tau235. Among these, p-tau217 has emerged as a superior and reliable marker of amyloid positivity and, thus, AD in terms of accuracy of diagnosis and ability for early prognosis. In this narrative review, we aim to elucidate the utility of p-tau217 as an AD marker, exploring its underlying basis, clinical diagnostic potential, and relevance in clinical care and trials.

## 1. Introduction

**Figure 1:**
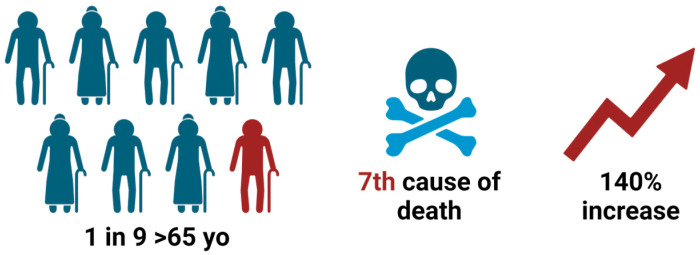
The significance of Alzheimer’s disease. Created with BioRender.com.

Alzheimer’s disease (AD) is a neurodegenerative disorder characterized by amyloid plaques and neurofibrillary tangles [1]. As the most common cause of dementia, AD affects 1 in 9 Americans aged 65 and older and was the seventh leading cause of death in the United States in 2021 [2] (Figure 1). While deaths from stroke and heart disease decreased from 2000 to 2021, deaths from AD increased more than 140% during this period, and AD is projected to affect 13.8 million Americans by 2060 [2] (Figure 1). Given that AD begins 20 years or more before symptom onset, there is a substantial window in which medical interventions may alter the disease progression [2]. Reliable biomarkers are needed for early detection of preclinical AD to delay or prevent the development of clinical AD.

Although the ultimate confirmation of AD diagnosis is based on postmortem examination of brain tissue for amyloid plaques consisting of amyloid-beta (Aβ) peptides and neurofibrillary tangles containing phosphorylated tau (p-tau), in vivo diagnosis can be achieved using cerebrospinal fluid (CSF) or neuroimaging biomarkers [1,3]. However, these methods are limited by the invasiveness of lumbar punctures and the high cost and specialized facilities required for positron emission tomography (PET) imaging [4]. In contrast, blood-based biomarkers are easily accessible and available at a fraction of the cost of neuroimaging, which allows the expansion of access to AD biomarker research and clinical care [4,5]. Among various plasma amyloid and tau markers, phosphorylated tau at threonine 217 (p-tau217) is one of the emerging tau markers that has repeatedly shown more promise than other p-tau isoforms as a marker of AD pathology. This review evaluates the evidence for p-tau217 as a peripheral biomarker of AD, compares it to other phosphorylated tau biomarkers, explores its relationship with imaging methods, and examines its utility in AD diagnosis and prognosis.

Current literature overwhelmingly focuses on another p-tau isoform, p-tau181. A literature search of articles published between 2020 and 2023 revealed 458 publications mentioning p-tau 181 and only 69 publications mentioning p-tau217. However, there have been studies suggesting that p-tau217 is superior to p-tau181 in comparisons such as correlation with tau-PET measures and differentiating between AD and non-AD states [6,7]. Thus, it appears that p-tau217 may be an understudied and underappreciated potential clinical tool for AD care. This study aims to address this discrepancy in focus by synthesizing current knowledge on p-tau217 by asking how p-tau217 compares to other AD biomarkers in performance. The hypothesis is that p-tau217 performs at parity, if not better, than p-tau181 and other p-tau isoforms.

## 2. AD Pathology and Biomarkers

**Figure 2:**
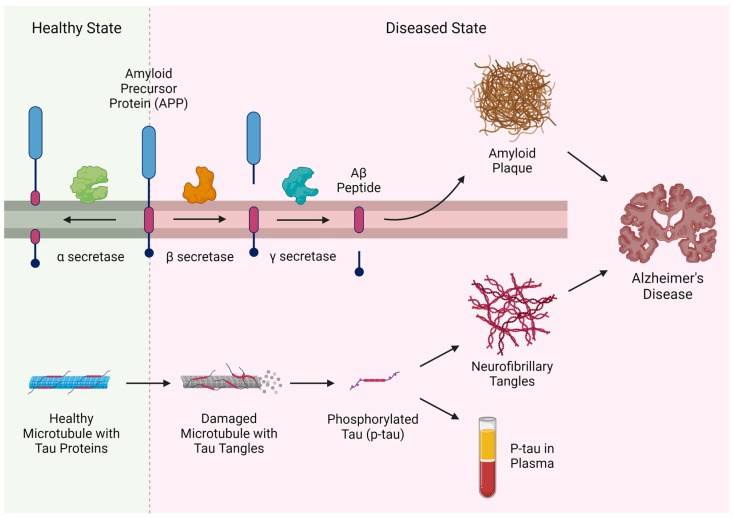
The pathophysiological process of Alzheimer’s disease. Created with BioRender.com.

AD is characterized by the presence of extracellular Aβ plaques and intracellular neurofibrillary tangles, composed of proteins formed by the aberrant cleavage of transmembrane amyloid precursor protein (APP) and microtubule-associated tau protein, respectively [1,8] (Figure 2). The major peptide in amyloid plaques is the amyloid β peptide (Aβ) which, as a hydrophobic molecule, is prone to aggregation [9]. Aβ is a proteolytic fragment of APP, which is a transmembrane protein commonly found within neurons and involved in synapse generation [10]. APP can be cleaved in three distinct locations by various secretases which either produce Aβ or prevent its generation. β-secretase cleaves APP at the N-terminal of the Aβ domain, while γ-secretase cleaves it at the C-terminal of the Aβ domain [10]. Therefore, cleavage of APP by β- and γ-secretase produces the insoluble Aβ peptide that can accumulate to form plaques [10,11]. In contrast, α-secretase cleaves APP within the Aβ domain and thus prevents the formation of complete Aβ and protects against AD [10,11]. Amyloid plaques are thought to disrupt synaptic communication, as well as trigger a local inflammatory response [12,13]. On the other hand, neurofibrillary tangles are thought to result from the breakdown of neuron microtubules [14]. Tau is a protein associated with microtubules that becomes hyperphosphorylated in the event of AD, forming p-tau [14]. In this form, p-tau dissociates from microtubules, aggregates intracellularly, and weakens microtubules, which in turn leads to a breakdown of axonal transport and even neuronal apoptosis [15]. The soluble building blocks of Aβ plaques and neurofibrillary tangles interact synergistically to drive neuronal death and synaptic dysfunction [1]. A growing body of evidence has suggested that Aβ functions upstream of tau in AD pathogenesis while tau exacerbates Aβ toxicity [16,17,18]. Given that soluble toxic Aβ and tau spread throughout the brain by prion-like mechanisms, with misfolded proteins converting their harmless counterparts into pathological forms, detection of these soluble peptides before they become observable plaques and tangles is essential for the early diagnosis and interventions of AD pathogenesis [19].

AD has historically been diagnosed based on cognitive and behavioral symptoms, but it suffers from poor correlation between clinical and postmortem neuropathological diagnosis [20,21]. More recently, AD has been classified using the AT(N) system based on core AD pathophysiological features, namely Aβ (A), tau (T), and neurodegeneration (N) [22,23]. Aβ markers include Aβ-PET and CSF Aβ42 or the ratio of Aβ42 to Aβ40 (Aβ42/Aβ40), while tau markers include tau-PET and CSF tau phosphorylated at various sites [23]. Markers of neurodegeneration include CSF total tau (t-tau), CSF neurofilament light chain (NfL), and hippocampal atrophy and/or cortical thinning determined by magnetic resonance imaging [23]. Newer studies have shown that blood-based markers may serve as less invasive and more affordable alternatives to neuroimaging and CSF biomarkers.

## 3. Tau as a Biomarker

Hyperphosphorylation of tau proteins is a characteristic pathological change in AD. There are many sites on the tau protein that may be phosphorylated at various disease stages from preclinical AD to mild cognitive impairment due to AD (MCI-AD) to clinical AD [24,25,26,27]. P-tau217, p-tau181, and p-tau231 have all been observed in neurofibrillary tangles and have been detected around Aβ plaques in mice with Aβ plaques without neurofibrillary tangles but not in mice with elevated Aβ without Aβ plaques, suggesting that these p-tau molecules are markers of postsynaptic pathology around Aβ plaques [28]. P-tau181 has been extensively studied and has seen clinical applications such as the use of CSF p-tau181 in the prediction of cognitive decline in patients with MCI [29]. However, p-tau217 has been suggested to perform better as a biomarker than p-tau181, with p-tau217 being more closely associated with AD severity [6]. P-tau217 accumulation may differentiate AD from related diseases, as higher p-tau217 has been observed in the brains of those with AD than those with primary age-related tauopathy or other non-AD tauopathies [30]. In addition, the spread of p-tau217 in postmortem brain tissue has shown a correlation with antemortem plasma p-tau217 in those with amyloid plaques but not in those without, suggesting that plasma p-tau217 may reflect the presence of Aβ plaques [30].

### Tau in Distinct Brain Regions

Hyperphosphorylation of tau has also been observed to show a differential association with neuronal loss from distinct brain regions. For example, nucleus basalis of Meynert (nbM) neuronal density was not correlated with p-tau concentrations, while lower locus coeruleus neuronal density correlated with higher p-tau217 [31]. In addition, norepinephrine depletion has been associated with increased p-tau217 and dose-dependent working memory deficits [32]. Together, these findings suggest that norepinephrine function may play a role in hyperphosphorylation of tau [32]. In the nbM, increased APP has been found to correlate with neurofibrillary tangle formation [33]. Furthermore, it has been found that 59% of the variability in plasma p-tau217 in the nbM can be predicted by a combination of global neuropathologic scales, especially tau and Aβ. This contrasts with just 31% of plasma p-tau181 variability being predicted by the same scales [31]. These data suggest that in the nbM, tau and amyloid β pathological processes are closely related to p-tau217 formation.

P-tau217 is also unique among tau biomarkers in that it has been found to be persistently associated with the CA1 region of the hippocampus, particularly within neurofibrillary tangles and granulovacuolar bodies (GVBs) [30]. GVBs are prominent among pyramidal neurons in the hippocampus and associate with the severity of tauopathies and Aβ pathology, spreading with tau pathology from the entorhinal cortex to the neocortex, hypothalamus, amygdala, and frontal and parietal cortices [34]. GVBs also correlate directly with neuronal cell loss and neurofibrillary tangle formation [34,35]. Taken together, p-tau217 may serve as a strong indicator of early-stage AD changes in the brain and may be more specific and better suited for clinical use than other tau biomarkers. 

## 4. P-tau217 in CSF and Plasma

### 4.1. CSF P-tau217

The wide array of tau markers with different phosphorylation sites requires the identification of a specific p-tau biomarker that exhibits the highest accuracy in AD. CSF studies have suggested that p-tau217 may be the best tau biomarker for AD [36,37,38,39]. Levels of p-tau217, p-tau181, and p-tau231 in CSF have all been observed to increase in both prodromal AD and AD dementia [36,37,38]. Among these, p-tau217 showed the greatest change between non-AD controls and AD patients, with a 13-fold increase compared to a 1.9- to 5.4-fold increase for the other p-tau variants [36]. CSF p-tau217 has also shown a correlation with markers of AD, including medial temporal lobe atrophy, cognitive performance, and Aβ burden [40]. Of all p-tau analytes, p-tau217 showed the strongest correlation with Aβ-PET and tau-PET status, two highly reliable neuroimaging methods used to evaluate AD [36,41]. Overall, p-tau217 had the highest accuracy in distinguishing AD from non-AD as well as predicting Aβ-PET and tau-PET positivity [36,38,39]. The higher increase in CSF levels and its strong correlation with other measures of AD suggest that CSF p-tau217 may be a better AD biomarker than other CSF p-tau biomarkers.

### 4.2. Plasma P-tau217

Plasma p-tau217 has also been demonstrated as a robust and accurate biomarker for AD with the advent of improvements in detection technology. Several p-tau isoforms like p-tau217, p-tau181, p-tau205, p-tau231, and p-tau235 have been observed in plasma in clinically, radiologically, and pathologically diagnosed AD [42,43,44,45,46,47]. In addition to their increased concentrations, plasma p-tau217, p-tau181, and p-tau231 all showed correlation with amyloid-PET status and predicted amyloid positivity [4,44,47,48,49]. Certain p-tau species such as 217 and 181 showed strong correlations in their CSF and plasma levels [47,50]. This correlation has been the strongest in p-tau217, suggesting that plasma p-tau217 may serve as a less invasive alternative to its counterpart in CSF [47,50]. Similar to its levels in CSF, plasma p-tau217 showed a greater increase than that of p-tau181, 4.2 and 1.7 fold, respectively, in amyloid-positive individuals [43]. Additionally, plasma p-tau217 detected amyloid and tau pathology with comparable accuracy to CSF p-tau217, while plasma p-tau181 and p-tau231 performed worse when predicting amyloid-PET status with respect to their CSF counterparts [51,52]. 

Studies comparing different plasma tau isoforms have suggested that p-tau217 may have the strongest predictive value due to its better dynamic range, discrimination of tau-PET positivity, and diagnostic performance compared to other p-tau biomarkers [53]. Stronger correlations have been reported between amyloid- and tau-PET status and plasma p-tau217 than any other isoform [41,46,54]. P-tau217 better identified amyloid-positivity and distinguished tau-positivity with higher accuracy [26,41,43,46,55]. P-tau217 has been shown to discriminate high and intermediate amyloid-PET scans with higher accuracy than p-tau181 [56]. Furthermore, p-tau217 was found to contribute to the identification of both amyloid and tau status, whereas p-tau231 was only found to contribute to the detection of tau positivity [57]. Notably, p-tau217 alone was noninferior to combining p-tau217 with Aβ42/Aβ40 when predicting Aβ positivity [58]. In summary, plasma p-tau217 has been shown to be noninferior to CSF p-tau217 while being superior to other plasma p-tau isoforms in the identification of Aβ- and tau-PET status.

## 5. P-tau217 as an AD Biomarker

### 5.1. Clinical AD

In addition to correlations with amyloid- and tau-PET status, plasma p-tau217 has been shown to differentiate pathologically diagnosed AD from non-AD with significantly higher accuracy than p-tau181, with an area under the curve (AUC) of 0.88–0.90 compared to p-tau181’s AUC of 0.72–0.81 [26,59,60]. Only plasma p-tau217 was able to differentiate between all levels of AD neuropathological change (none, low, intermediate, and high), while p-tau181 and p-tau231 only differentiated between intermediate and high AD neuropathology [60]. Higher concentrations of p-tau217 in antemortem plasma and CSF as well as postmortem brain samples have been reported in clinical AD and MCI compared to cognitively unimpaired (CU) individuals [40,61]. Among six phosphorylation sites, antemortem plasma p-tau217 showed the greatest increase in autopsy-confirmed AD compared to non-AD participants and p-tau217 showed the strongest association with both Braak and Consortium to Establish a Registry for Alzheimer’s Disease (CERAD) stages [59].

Limited data exist on the p-tau217 levels in atypical or non-amnestic presentations of AD such as logopenic variant primary progressive aphasia (lvPPA) or posterior cortical atrophy (PCA). One study found an average plasma p-tau217 concentration of 0.72 ± 0.4 pg/mL in the amnestic presentation of AD, 0.82 ± 0.2 pg/mL in lvPPA, and 0.80 ± 0.3 pg/mL in PCA compared to 0.17 ± 0.1 pg/mL in normal controls [46]. While no statistical tests were performed to compare the p-tau217 levels in different AD presentations, it appears that lvPPA and PCA show elevated p-tau217, similar to the typical presentation of AD.

### 5.2. Preclinical AD

Compared to other p-tau isoforms, p-tau217 showed larger increases at earlier disease stages, with notable elevations starting at PET-based Braak stage II and plateauing at stage IV [24,47]. In contrast, p-tau181 demonstrates greater increases at later stages [24]. P-tau217 levels increase before detectable tau aggregation, and p-tau217 can distinguish amyloid-positive tau-negative individuals from non-AD controls [47]. This early rise in p-tau217 is hypothesized to be due to its unique close association with the CA1 region of the hippocampus and localization with GVBs [30]. The formation of GVBs is driven by neuronal damage and neurofibrillary tangle formation. Interestingly, GVBs have been found to participate in exosomal secretion, disposing of cellular waste, such as p-tau217, extracellularly [62]. It is hypothesized that the secretion of GVBs in response to early-stage AD neurofibrillary tangle formation contributes to p-tau217’s unique early staging [30].

The early rise in p-tau217 levels during the course of AD renders it an important biomarker of preclinical AD. For example, in an inherited form of AD, p-tau217 levels were found to rise 21 years before symptom onset, while p-tau181 and p-tau205 levels increased 19 and 13 years, respectively, before symptom onset [25]. Elevated p-tau217 has also been reported in preclinical AD up to 20 years before MCI-AD onset [26,27]. In CU individuals, plasma p-tau217 correlates with amyloid- and tau-PET status as well as with CSF Aβ42/Aβ40 [58,63]. Additionally, p-tau217 independently identified Aβ status and was not influenced by demographic factors in the detection of Aβ positivity in CU individuals [27,57]. These findings underscore the potential of p-tau217 as a highly sensitive and specific biomarker for the early detection, diagnosis, and monitoring of preclinical AD.

### 5.3. Role of P-tau217 in AD Prognosis

The early rise of p-tau217, prior to detectable tau aggregation or symptom onset, also makes it a promising candidate for the monitoring of AD progression. Baseline p-tau217 has been found to be associated with longitudinal Aβ-PET increases in those without overt Aβ pathology at baseline [44]. In addition, p-tau217, but not p-tau181 or p-tau231, showed an annual increase only in Aβ-positive individuals, with the greatest change in Aβ-positive tau-positive individuals [52,64]. With disease progression, p-tau217 area fraction has been shown to correlate strongly with amyloid-beta and neurofibrillary tangle brain load [30].

Not only is p-tau217 associated with amyloid plaque and neurofibrillary tangle accumulation, but it has also been shown to correlate with cognitive measures in both clinical and preclinical AD [38,39,63]. Among Aβ-positive participants, p-tau217 predicted global cognition, memory, language, executive function, visuospatial skills, and cortical thickness with higher accuracy than p-tau181 [65]. Longitudinal increases in p-tau217 have shown an association with worsening cognition and brain atrophy [66]. 

P-tau217 may be useful in the prediction of progression from preclinical AD or MCI to clinical AD. Both higher baseline p-tau217 and more rapid longitudinal increase in p-tau217 levels have shown an association with future development of clinical AD in individuals with MCI [66,67,68]. In contrast, no longitudinal change in p-tau217 levels was observed in those with MCI who did not later develop clinical AD [66]. The combination of p-tau217 with memory, executive function, and *APOE* genotype has been shown to further increase the predictive accuracy of progression from MCI to clinical AD [68]. Among those with MCI, Aβ-positive participants have been found to have an accelerated increase in p-tau217 compared to Aβ-negative individuals, who did not show a longitudinal change in p-tau217 levels [66].

Among CU individuals, associations have been found between p-tau217 and worse clinical progression, as well as decreased cortical thickness [64,65]. A more rapid longitudinal increase in p-tau217 has been observed in Aβ-positive CU participants compared to Aβ-negative CU participants, who showed no significant longitudinal change in p-tau217 levels [66]. In CU individuals, p-tau217 was associated with an increased risk of MCI development in Aβ-positive participants, while no such association was observed in their Aβ-negative counterparts [69]. Across Braak stages, lower postmortem p-tau217 was associated with better baseline memory [70]. In contrast, higher p-tau217 at preclinical stages was associated with worse cognitive trajectories [63]. Taken together, these results suggest that higher baseline levels and faster longitudinal increases in p-tau217 are associated with more severe Aβ pathology and cognitive impairments while also predicting worse clinical progression.

### 5.4. Role of P-tau217 in Differentiating AD from Non-AD Dementia

Tau biomarkers have been shown to differentiate tauopathies [71]. CSF p-tau has been observed to increase in AD but not in non-AD dementias including progressive supranuclear palsy and corticobasal syndrome [4,72,73]. In addition, individuals with Lewy body dementia showed lower CSF t-tau and p-tau levels than those with AD [74]. Similarly, lower t-tau and higher p-tau to t-tau ratios (p/t-tau) have been observed in frontotemporal dementia (FTD) than in AD [75]. In fact, FTD is associated with lower p/t-tau compared to non-AD controls [76].

P-tau217 has also shown utility in distinguishing AD from other neurodegenerative diseases [26,67]. Reduced plasma p-tau217 extracellular vesicles (EVs) have been observed in AD compared to non-AD controls while smaller p-tau217 EVs have been found in AD compared to non-AD dementia [77]. A model combining patient age with number and size of EVs carrying p-tau217 discriminated AD from non-AD controls and non-AD dementia with high accuracy [77]. While elevated p-tau217 has been observed in AD and other neurodegenerative diseases, p-tau217 distinguished AD from disorders such as primary progressive aphasia (specifically the nonfluent and semantic variants), frontotemporal dementia, progressive supranuclear palsy, and corticobasal degeneration with 90% accuracy, while p-tau181 showed a 78% accuracy [78]. In another study, p-tau217 performed better than p-tau181 when discriminating clinically diagnosed and pathology-confirmed AD from frontotemporal lobar degeneration [46]. 

## 6. Screening Assays for P-tau Analytes

Blood-based biomarkers for AD have generally been preferred to ones in CSF because of their accessibility. Plasma Aβ42/Aβ40 is correlated with pathologically identified Aβ in the brain, while plasma NfL is associated with neurodegeneration in AD [60,79]. Plasma p-tau is especially promising due to its large increase in AD, strong correlation with established diagnostic methods, and ability to differentiate AD from other neurodegenerative disorders [4,53,67,80]. However, biomarkers of AD are inherently more abundant in the CSF due to its constant contact with the brain compared to biomarkers in plasma, leading to the historic difficulty of blood-based AD biomarker development [60]. Nevertheless, technological developments in recent decades, such as immunoprecipitation and mass spectrometry, and improvements in enzyme-linked immunosorbent assays (ELISA) have greatly increased the accuracy and viability of blood-based biomarkers.

P-tau levels detected via ELISA had been the mainstay of p-tau measurements, with CSF p-tau231 having a reported sensitivity of 90.2% and specificity and 80.0% in discriminating between AD and non-AD disorders [81]. With advances in immunoassay technology, the sensitivity and specificity were increased in CSF p-tau217 to 91% and 91% for discriminating between AD and non-AD disorders [6]. Plasma biomarkers of AD have been inferior to the CSF and neuroimaging methods due to technological limitations of assays to quantify significantly lower concentrations of markers in plasma [80]. 

Developments within the last decade have increasingly turned to mass spectrometry to quantify p-tau levels. Barthelemy et al. utilized mass spectrometry combined with nano-flow capillary liquid chromatography to analyze both plasma and CSF p-tau peptides [47,82]. Indeed, a comparison between multiple immunoassay methods and the mass spectrometry method outlined by Barthelemy found that mass spectrometry performed significantly better [83]. Mass spectrometry performed better than immunoassay methods in detecting abnormal Aβ status, progression to AD, and showed the strongest correlation between plasma and CSF p-tau [83]. With regard to p-tau217, mass spectrometry was found to have the best performance out of any other p-tau biomarker in detecting abnormal Aβ status and had strong predictive value for identifying patients with MCI and abnormal Aβ levels, and those who progressed to AD [83]. Taken together, these results imply that mass spectrometry methods for p-tau detection, specifically p-tau217, may have strong future roles in AD detection and prognosis, edging out older immunoassay methods in terms of predictive value and accuracy.

Therriault et al. found that recent new developments in ultrasensitive immunoassays for p-tau perform comparably or better than previously described liquid chromatography–mass spectrometry methods [84]. Diagnostic performance between the two methods was similar for p-tau217, but mass spectrometry for p-tau181 and p-tau231 was inferior to immunoassay methods with regard to association with amyloid-PET and tau-PET [84]. 

More recently, a novel chemiluminescent enzyme immunoassay (CLEIA) to detect p-tau analytes has been developed. While ELISA measures optical density, CLEIA measures relative light units. In comparison, CLEIA methods have higher sensitivity and are less time-consuming compared to ELISA but are more costly [85]. This, too, is true for p-tau: CLEIA detection of p-tau181 in CSF was found to be more accurate than methods using ELISA [86]. CLEIA kits for p-tau217 have been developed and have shown high accuracy in discerning AD from other neurodegenerative diseases [87]. Literature-reported sensitivities, specificities, and AUC for discerning between AD and non-AD controls for p-tau analytes in plasma and CSF can be found in Table 1. With recent advances, ELISA, mass spectrometry, and CLEIA methods have demonstrated consistent, similar testing characteristics across all analytes. However, p-tau217 tests perform notably better than those measuring p-tau181 or p-tau231, once more suggesting its superiority in utility for clinical AD testing. 

In summary, developments in mass spectrometry and immunoassay methods have created near parity in their diagnostic performance, notably in the detection of p-tau217, with each having strengths and limitations. Mass spectrometry is able to provide absolute quantification of p-tau levels, whereas immunoassays cannot [84]. Furthermore, mass spectrometry is able to quantify multiple analytes, at the cost of decreased sensitivity, whereas immunoassays measure only a single prespecified analyte [80]. The ability to measure and quantify various p-tau analytes may prove vital to the prognosis and measurement of disease progression. As previously mentioned, distinct p-tau markers have been found to exist at different concentrations depending on the timescale of disease, and the ability to examine all levels of p-tau in one single assay may provide the best clinical picture [88]. Should a clinician wish to view p-tau217 levels specifically, immunoassay methods may be chosen instead to mitigate the slower result times and higher machine costs of mass spectrometry. Although CLEIA methods for p-tau are more recent compared to established ELISA and mass spectrometry assays, they represent a possibly quicker and more accurate alternative, although their current cost and novelty may hold them back from general use. For the widespread adoption of p-tau as a tool for AD diagnosis and monitoring in the clinic, however, universal cutoffs for p-tau levels are still required [46]. Additional research is needed to establish these values for clinical use.

**Table 1:** Reported sensitivities, specificities, and AUC of ELISA, mass spectrometry, and CLEIA detection methods for the p-tau analytes p-tau181, p-tau217, and p-tau231.

	ELISA	Mass Spectrometry	CLEIA
p-tau181 (plasma)	Sen: 86.6% [89]	Sen: No data	Sen: No data
	Spe: 80.0% [89]	Spe: No data	Spe: No data
	AUC: 0.889 [89]	AUC: 0.98 [47]	AUC: 0.910 [90]
p-tau217 (plasma)	Sen: 95% [91]	Sen: No data	Sen: No data
	Spe: 94% [91]	Spe: No data	Spe: No data
	AUC: 0.98 [91]	AUC: 0.98 [47]	AUC: 0.952 [87]
p-tau231 (plasma)	Sen: 81.2% [5]	Sen: No data	Sen: No data
	Spe: 93.3% [5]	Spe: No data	Spe: No data
	AUC: 0.94 [5]	AUC: 0.834 [59]	AUC: No data
p-tau181 (CSF)	Sen: 91.8% [89]	Sen: No data	Sen: 90% [86]
	Spe: 90.5% [89]	Spe: No data	Spe: 90% [86]
	AUC: 0.954 [89]	AUC: 0.95 [47]	AUC: 0.98 [86]
p-tau217 (CSF)	Sen: No data	Sen: No data	Sen: No data
	Spe: No data	Spe: No data	Spe: No data
	AUC: 0.95 [41]	AUC: 1.00 [47]	AUC: No data
p-tau231 (CSF)	Sen: No data	Sen: No data	Sen: No data
	Spe: No data	Spe: No data	Spe: No data
	AUC: 0.88 [41]	AUC: 0.9873 [92]	AUC: No data

## 7. Role of P-tau217 in Clinical Trials

Plasma p-tau217 may help improve future clinical trials by enhancing participant selection, distinguishing progressors from non-progressors, and monitoring the effects of treatments. Plasma p-tau217 may be useful for the identification of preclinical AD among CU individuals. Given its strong correlation with tau-PET status, Aβ-PET status, and CSF p-tau concentrations, plasma p-tau217 may be used to pre-screen clinical trial participants so that only individuals with high plasma p-tau217 receive PET scans and/or CSF collections. Though this may help to reduce cost and the number of invasive procedures during screening, clinical trials on anti-tau and anti-Aβ treatments generally need to monitor target engagement throughout the study, so PET scans may still be necessary during enrollment. The TRAILBLAZER-ALZ 3 (NCT05026866) trial in a large sample of CU older adults is unique in being the first to use plasma p-tau217 as the only enrollment criterion, while Aβ-PET is not performed in any part of enrollment or treatment monitoring [93].

Given the slow progression from preclinical to clinical AD, identification of individuals who will later develop clinical AD versus those who will remain stable may be important to shorten clinical trials and reduce the sample size needed to detect the effects of potential medications. Plasma p-tau217 has shown promise in its ability to predict cognitive decline and progression to clinical AD in both MCI and CU individuals and therefore may serve another important role in the selection of clinical trial participants.

Considering the ease of plasma biomarker collection, p-tau217 may also be used to monitor the effects of anti-tau and anti-Aβ treatments more frequently. In the concluded TRAILBLAZER-ALZ (NCT03367403) trial, a significant decrease in plasma p-tau217 was observed compared with placebo after just 12 weeks of treatment and continued throughout the 76-week duration of the study [49]. Furthermore, lower plasma p-tau217 levels correlated with the reduction in tau- and Aβ-PET in the treatment group, suggesting that plasma p-tau217 may be useful for monitoring brain neurofibrillary tangles and amyloid plaque accumulation during treatment [94,95].

In addition to its role in enrollment and monitoring of treatments, p-tau217 itself may serve as a potential target for treatment in the future. For example, monoclonal antibodies against p-tau217 have been found to reduce tau aggregation, block apoptosis-associated neuronal loss, decrease brain atrophy, reverse cognitive deficits, and improve motor function in tauopathic mice [40].

## 8. Challenges and Limitations

**Figure 3:**
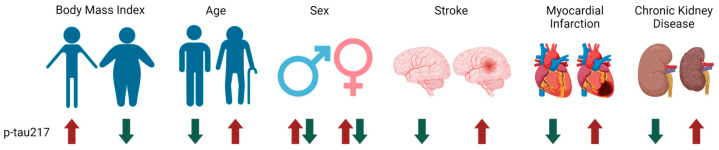
The effects of various demographic factors and comorbidities on plasma p-tau217 concentrations. The up arrows (↑) indicate an increase in levels, the down arrows (↓) point to a decrease in levels, and a combination of up and down arrows (↑↓) suggests an indeterminate effect. Created with BioRender.com.

Plasma p-tau levels may show variations within and between individuals based on factors unrelated to AD, such as body mass index (BMI), age, or sex, which poses a potential limitation in their utility in AD diagnosis and prognosis (Figure 3). A study found a 10.3% within-subject variation in p-tau217, while p-tau231 showed a 6.3% variation and p-tau181 showed a 16.7% variation [96]. Similarly, p-tau217, p-tau231, and p-tau181 varied by 21.1%, 17.2%, and 25.7%, respectively, among healthy individuals [96]. Higher p-tau217 has been observed in those with lower BMI [50]. When comparing biomarker levels among participants aged 30 to 98 years, an increase in plasma p-tau217 with age can be observed starting at ages 65 to 69 [97]. However, when participants were grouped by amyloid-PET status, the increase in p-tau217 with age was 12 times faster in amyloid-positive than amyloid-negative participants [97]. Mixed results have been reported regarding the relationship between sex and p-tau217 levels. Among CU individuals, males showed significantly higher p-tau217 than females, whereas males and females in the MCI or dementia groups showed no differences in p-tau217 levels [97]. No sex differences were found in the increase in p-tau217 with age in participants with or without *PSEN1* mutations, the most common cause of familial AD [98,99]. However, female *PSEN1* mutation carriers showed higher p-tau217 than male carriers [98]. In CU *PSEN1* mutation carriers, males had worse cognitive performance than females as p-tau217 concentrations increased, while no interaction between age and sex was observed in cognitively impaired mutation carriers [98].

Certain comorbidities such as stroke, myocardial infarction (MI), or chronic kidney disease (CKD) may also increase the concentrations of plasma p-tau and therefore decrease their utility in the diagnosis of AD (Figure 3). Increased p-tau217 levels have been reported in individuals with a history of stroke compared to those without [97]. Similarly, those with a history of MI have shown higher p-tau217 than those without a history of MI [97]. Reduced kidney function and CKD were associated with increased p-tau217 in both cognitively impaired and unimpaired individuals [50,100]. More alarmingly, p-tau level differences between CKD and non-CKD individuals have been observed to be comparable to differences between amyloid-positive and amyloid-negative participants [97]. Adjusting for creatinine and BMI, however, has been found to improve the discrimination by plasma p-tau217 of stable CU and MCI individuals from those who later develop AD, especially in individuals with higher creatinine and BMI [50]. In addition, the ratio of phosphorylated to unphosphorylated tau (pT217/T217) could more accurately reflect pathological changes in AD. pT217/T217 was not correlated with kidney dysfunction in those with MCI and only weakly correlated in CU individuals [100]. Furthermore, in both MCI and CU cohorts, pT217/T217 showed a much larger difference between amyloid-positive and amyloid-negative participants than between CKD and non-CKD individuals [100]. Therefore, adjusting for creatinine and BMI or the use of pT217/T217 may serve to mitigate the variations in plasma p-tau217 due to factors unrelated to AD.

Another factor of the viability of p-tau217’s use as a clinical tool will be its cost. As per the Brown University Center for Alzheimer’s Disease Research, a current Simoa^®^ plasma p-tau217 assay is listed to cost USD 194 commercially. Comparisons of prices with other assays as well as imaging modalities can be found in Table 2. It must be noted that the prices of these assays do not include various extraneous hospital fees that may be incurred by the patient. Prices, especially for imaging studies, undoubtedly fluctuate greatly between institutions as well. Despite these limitations, the cost of plasma and CSF biomarker determination is still much lower than that of an amyloid-PET scan. As Alzheimer’s testing technology advances, it is hoped that the cost to the healthcare system and to the patient will decrease.

## 9. Conclusions

Hyperphosphorylation of tau is a hallmark pathological change in AD present around Aβ plaques and in neurofibrillary tangles. Among the many phosphorylated tau biomarkers, p-tau217 has attracted special attention in recent years due to its utility in AD diagnosis and prognosis. Its increase in the early stages of AD makes p-tau217 a useful biomarker for the diagnosis and prognosis of both clinical and preclinical AD. Elevated p-tau217 can be observed more than 20 years prior to symptom onset and is strongly associated with later diagnosis of AD. The field of p-tau detection and quantification has developed rapidly in the past several years, making less invasive and more cost-effective blood biomarkers for AD increasingly viable. Research on plasma p-tau217 has shown it to be an accurate and unique biomarker for AD and AD progression. However, further research is needed for its formal adoption as a clinical tool.

## Figures and Tables

**Table 2:** Selected reference costs of AD laboratory tests and imaging. Simoa^®^ and SMC™ assays are usable on both plasma and CSF samples. HD-X: HD-X Automated Immunoassay Analyzer; GFAP: glial fibrillated acidic protein; NF-light: neurofilament light.

Procedure	Cost
Simoa^®^ pTau217 for HD-X	USD 194 [101]
Simoa^®^ pTau181 for HD-X	USD 172 [101]
Simoa^®^ pTau231 for HD-X	USD 141 [101]
Simoa^®^ GFAP for HD-X	USD 145 [101]
Simoa^®^ Neurofilament Light (NfL) for HD-X	USD 207 [101]
Simoa^®^ Neurology 4-Plex E for HD-X (AB40, AB42, GFAP, NF-light)	USD 401 [101]
Simoa^®^ Neurology 3-Plex A for HD-X (AB40, AB42, Tau)	USD 338 [101]
SMC™ Amyloid Beta 40	USD 137 [101]
SMC™ Amyloid Beta 42	USD 137 [101]
Amyloid-PET scan	USD 6487 [102]

## Abbreviations

Aβ	amyloid-beta
Aβ42/Aβ40	ratio of Aβ42 to Aβ40
AD	Alzheimer’s disease
APP	amyloid precursor protein
AUC	area under the curve
BMI	body mass index
CERAD	Consortium to Establish a Registry for Alzheimer’s Disease
CKD	chronic kidney disease
CLEIA	chemiluminescent enzyme immunoassay
CSF	cerebrospinal fluid
CU	cognitively unimpaired
ELISA	enzyme-linked immunosorbent assay
EV	extracellular vesicle
FTD	frontotemporal dementia
GVB	granulovacuolar body
lvPPA	logopenic variant primary progressive aphasia
MCI-AD	mild cognitive impairment due to AD
MI	myocardial infarction
nbM	nucleus basalis of Meynert
NfL	neurofilament light chain
PCA	posterior cortical atrophy
PET	positron emission tomography
p-tau	phosphorylated tau
p-tau217	phosphorylated tau at threonine 217
p/t-tau	p-tau to t-tau ratio
pT217/T217	ratio of phosphorylated to unphosphorylated tau
t-tau	total tau
